# Advances in Food and Byproducts Processing towards a Sustainable Bioeconomy

**DOI:** 10.3390/foods8090425

**Published:** 2019-09-19

**Authors:** Nikolaos Kopsahelis, Vasiliki Kachrimanidou

**Affiliations:** 1 Department of Food Science and Technology, Ionian University, 28100 Argostoli, Kefalonia, Greece; 2 Department of Food and Nutritional Sciences, University of Reading, Reading RG6 6AP, UK

**Keywords:** biorefineries, circular economy, bioeconomy, food processing, food biotechnology, bioprocesses, bioactive compounds, food waste valorization, sustainability

The bioeconomy concept was initially focused on resource substitution, aiming to mitigate the depletion of fossil resources and confer an alternative approach for resource utilization. Within this context, the production of biomass from diversified resources, along with its subsequent conversion, fractionation, and processing, was deemed of paramount importance. Thus, biotechnological and chemical routes along with process engineering were implemented for the production and marketing of food, feed, fuel, and fiber.

Nowadays, although resource substitution is still considered important, and regardless of the substantial accomplishments of chemical treatments for efficient biomass utilization, emphasis has shifted towards the biotechnological innovation perspective—to configure bioprocesses that will be included in the restructure of current facilities to develop consolidated processes, under the frame of transition to a circular model that will confer environmental sustainability.

Alongside this shift, global projections of food loss are estimated at one-third of the total quantity produced for human consumption, posing not only a sustainability issue related to food security but also a significant environmental concern. Likewise, food waste streams, derived primarily from fruits and vegetables, cereals, oilseeds, meat, dairy, and fish processing, constitute an abundant pool of complex carbohydrates, proteins, lipids, and functional compounds. Hence, the deployment of food waste streams as raw materials will encompass the formulation of added-value products that will be ideally reintroduced in the food supply chain to close the loop.

This Special Issue is devoted to the development of innovative and emerging food and byproducts processing methods, as a necessity for the sustainable transition to a bioeconomy era. Valorization, bioprocessing and biorefining of food-industry-based streams, the isolation of high added-value compounds, applications of the resulting bio-based chemicals in food manufacturing, novel food formulations, economic policies for food waste management, along with sustainability or technoeconomic assessment of processing methods constitute subject areas that need to be addressed.

More specifically, bioprocess design to valorize food-industry waste and byproducts streams should be initiated by characterizing the composition of the onset raw material with the aim of identifying the target end-products, whereas the generation of multiple high added-value products is a prerequisite for cost-effective processes to establish economic sustainability. On top of that, the feasibility of innovative processing could be sustained by encompassing food applications, driven by the constantly emerging consumers’ demand for functional foods with enhanced nutritional value. Equally, a growing awareness for bio-based and natural food components is being developed, thereby imposing challenges on the substitution of chemically derived ingredients with their natural counterparts.

Within this context, Papadaki et al. [1] evaluated the production of *Morchella* sp. as one of the most expensive mushrooms conferring high nutritional value, using agroindustrial starch-based substrates, specifically wheat grains, potato peels, and a mixture of both. Submerged and solid-state fermentations were employed to study biomass formation and polysaccharide content. In another study, Koliastasi et al. [2] presented the utilization of composted olive processing solid waste to extract emulsifiers, composed mainly of oligosaccharides and oligopeptides. The emulsifying capacity and stabilizing properties of the extracted emulsifiers indicated that valorization of olive processing waste through partial-composting might provide a novel, fast, and economic method of production of high added-value products. Likewise, Hou et al. [3] presented the effect of drying methods (dehydration freeze-drying) on the properties of heat-induced gels using porcine plasma protein powder.

Apart from applications in food formulation, high-value products as food packaging materials can also be obtained using renewable sources. A case examining the interaction of arabinoxylan films with soil and water was provided by Anderson and Simsek [4]. Wheat bran, maize bran, and dried distillers grain combined with glycerol or sorbitol were used to formulate arabinoxylan films, followed by the study of biodegradability, water solubility, and water vapor transmission rate.

The employment of bacterial, fungal, and yeast strains to yield added-value products via their proliferation on renewable substrates comprises the fundamental principle of bioprocess design. Likewise, enzymes entail a frequently studied end-product in fermentative bioconversions. Makanjuola et al. [5] used sorghum bran, a processing waste rich in starch, for the secretion of glucoamylase in submerged fermentations using the fungal strain *Aspergillus awamori*. Cultivation parameters were evaluated to obtain maximum enzyme production, and subsequently hydrolysis experiments were performed, resulting in a glucose-rich solution for further involvement in microbial fermentation.

Among the most studied bio-based products, microbial oil synthesized by oleaginous yeasts and fungi has attracted extensive research interest for the subsequent implementation for biodiesel production or the inclusion in food formulation. Patel et al. [6] studied the culture of *Rhodosporidium kratochvilovae* on clarified butter sediment waste for the production of single cell oil, demonstrating high lipid accumulation. The compliance with ASTM 6751 and EN 14214 international standards was also evaluated, indicating the potential use for biodiesel synthesis. In a similar study, Tsakona et al. [7] proposed the utilization of diversified mixed confectionery waste streams in a two-stage bioprocess to generate nutrient rich feedstock for the proliferation of *Rhodosporidium toruloides* DSM 4444 to generate microbial oil under various fermentation strategies. The fatty acid profile was altered based on the initial substrate, with oleic acid being the predominant one, thereby insinuating the production of tailor-made lipids targeting innovative food applications.

Ultimately, the transition to a bioeconomy era will be facilitated by designing consolidate bioprocesses that confer a holistic approach and focus on innovative end-applications, which could be integrated in existing manufacturing plants. A holistic and efficient resource utilization is unequivocally a prerequisite to consider within the development of fermentation bioconversions. For instance, the proposed biorefinery scheme using diversified mixed confectionery streams suggested the generation of value-added products that could be reintroduced in the food supply chain. In a similar manner, Lappa et al. [8] elaborated the most recent advances and approaches to benefit, from the complete exploitation of cheese whey stream. The proposed integrated biorefineries target the cost-effective production of novel value-added products to convey enhanced sustainability and incorporate a “zero waste” approach. Retrospectively, the design of consolidate bioprocesses towards a circular and bio-based economy should also take into account the founding pillars of economy, society, and environment. It is envisaged that this Special Issue will succeed in providing state-of-the-art advances in food and byproducts processing within the bioeconomy era.
